# Comparative physiological and biochemical mechanisms of salt tolerance in five contrasting highland quinoa cultivars

**DOI:** 10.1186/s12870-020-2279-8

**Published:** 2020-02-12

**Authors:** Zhi-Quan Cai, Qi Gao

**Affiliations:** 1 Department of Horticulture, Foshan University, Foshan, 528000 China; 2 CAS Key Laboratory of Tropical Plant Resources and Sustainable Use, Xishuangbanna Tropical Botanical Garden, Chinese Academy of Sciences, Mengla, 666303 China

**Keywords:** *Chenopodium quinoa*, Salt stress, Antioxidant enzyme, Growth, Inorganic ions, Organic solutes

## Abstract

**Background:**

*Chenopodium quinoa* Willd., a halophytic crop, shows great variability among different genotypes in response to salt. To investigate the salinity tolerance mechanisms, five contrasting quinoa cultivars belonging to highland ecotype were compared for their seed germination (under 0, 100 and 400 mM NaCl) and seedling’s responses under five salinity levels (0, 100, 200, 300 and 400 mM NaCl).

**Results:**

Substantial variations were found in plant size (biomass) and overall salinity tolerance (plant biomass in salt treatment as % of control) among the different quinoa cultivars. Plant salinity tolerance was negatively associated with plant size, especially at lower salinity levels (< 300 mM NaCl), but salt tolerance between seed germination and seedling growth was not closely correlated. Except for shoot/root ratio, all measured plant traits responded to salt in a genotype-specific way. Salt stress resulted in decreased plant height, leaf area, root length, and root/shoot ratio in each cultivar. With increasing salinity levels, leaf superoxide dismutase (SOD) activity and lipid peroxidation generally increased, but catalase (CAT) and peroxidase (POD) activities showed non-linear patterns. Organic solutes (soluble sugar, proline and protein) accumulated in leaves, whereas inorganic ion (Na^+^ and K^+^) increased but K^+^/Na^+^ decreased in both leaves and roots. Across different salinity levels and cultivars, without close relationships with antioxidant enzyme activities (SOD, POD, or CAT), salinity tolerance was significantly negatively correlated with organic solute and malondialdehyde contents in leaves and inorganic ion contents in leaves or roots (except for root K^+^ content), but positively correlated with K^+^/Na^+^ ratio in leaves or roots.

**Conclusion:**

Our results indicate that leaf osmoregulation, K^+^ retention, Na^+^ exclusion, and ion homeostasis are the main physiological mechanisms conferring salinity tolerance of these cultivars, rather than the regulations of leaf antioxidative ability. As an index of salinity tolerance, K^+^/Na^+^ ratio in leaves or roots can be used for the selective breeding of highland quinoa cultivars.

## Background

As a global issue, soil salinisation limits agricultural production because of its detrimental effect on seed germination, plant growth and crop yield [1, 2]. In order to tolerate soil salinization conditions, besides anatomical and morphological plasticity, plants have evolved multiple physiological mechanisms, e.g., osmotic adjustment, increased antioxidant response, accumulation or exclusion of ions, and ion homeostasis to maintain plant growth. For osmotic adjustment, inorganic ions (K^+^, Na^+^ and Cl^−^) and compatible organic solutes (e.g. soluble sugars, proline, glycine betaine, and polyamines) are the key osmolytes which play vital role to reduce cell water potential [1, 3]. However, the contribution to osmotic adjustment via accumulation of organic solutes under salt stress is still disputed, depending on the species/genotypes, duration and intensity of the stress, confounding effect of other factors, and also leaf and/or plant ages [4, 5].

Being a physiologically and genetically complex trait, salinity tolerance is associated with multiple subtraits (e.g. osmotic balance, ion homeostasis, and reactive oxygen species regulation), each having a complex and less understandable genetic basis [1]. Thus, genetic variation can be testified indirectly by measuring the responses of different species and/or genotypes, as well as by ontogenetic stages. As salt tolerant plants are rare (< 0.25% of flowering plants [6];) and the majority of main crops (rice, maize, etc.) belong to glycophytes, for growers, the most efficient way is the usage of alternate salt-adaptive crop species (obligatory halophytes), minimizing the harmful effects of salinity on crop’s growth and production.

Quinoa (*Chenopodium quinoa* Willd.) belonging to the family Amaranthaceae, a pseudo-cereal native to Andes, has provided nutrition and medicine for the local people over several thousands of years, owing to the high content of health-beneficial phytochemicals in seed [7]. Resistant to multiple abiotic stresses (e.g. drought, salinity, frost and poor soils), quinoa offers to be a promising crop to endure the increasing drought and salinity conditions under the global climatic change scenario [8]. Being cultivated in widely edapho-climatic conditions since about last 7000 years, the broad diversity has traditionally led to the classification of five quinoa ecotypes adapted to different native geographic environments: salares, highlands, inter-Andean valleys, yungas, and coastal lowlands [9]. Among them, although it is well-known that salares landraces have the highest salinity tolerance, extent of salinity tolerance of the highland ecotype, growing at high altitudes around Titicaca Lake, has received less attention. Serving as a putative model halophytic crop, quinoa displayed a wide degree of variability in salinity tolerance strategies based on its genome [10, 11]. Apart from the distinct anatomical features, i.e., salt bladders on both leaf adaxial and abaxial surfaces, salinity tolerance in quinoa plants was achieved through multiple strategies operating simultaneously, depending on the genetic background (ecotypes or genotypes) and the duration and intensity of the stress [12–14]. For instance, plant salt tolerant and ion homeostasis are maintained by membrane transporters, i.e., SOS1 (salt overly sensitive), NHX1 (Na^+^/H^+^ exchanger), H^+^-ATPase, HAK (high-affinity K^+^ transporter) and HKT (high-affinity K^+^ transporter) [1]. SOS1 controls the extrusion of Na^+^ and is also involved in the transport processes implicated in the xylem and phloem loading/unloading of Na^+^ in plants (long-distance transport) [1]. NHX-type antiporters in the tonoplast, mediating the compartmentation of Na^+^ in vacuoles, have a central role in establishing ion homeostasis and have been reported to increase the salt tolerance of various plants species [1, 4, 11]. Differential spatial and temporal expressions of these transporter genes synergistically regulate ion homeostasis by controlling Na^+^ transport systems at the tissue- and whole-plant levels under salt conditions. In four lowland genotypes of quinoa, the expression of two sodium transporter genes (i.e. CqSOS1 and CqNHX1) was differentially induced at different tissues (shoots vs. roots), and between genotypes (more salt-tolerant vs. less salt-tolerant genotypes) [13], suggesting that plant salt tolerance may depend upon different mechanisms of ion (Na and/or K) uptake/exclusion, translocation and compartmentation.

On the other hand, plants resource allocation between growth and stress tolerance is a major evolutionary constrain on plants. A basic tenet of plant ecophysiology is the growth-stress tolerance tradeoff proposed by the competitor – stress tolerator – ruderal (C-S-R) triangle theory, i.e., inverse relationships between the capacity of species/genotypes to grow when resources are abundant and its capacity to tolerate resource shortage [15, 16]. For instance, the morphological and physiological traits associated with low-light compensation points enable slow-growing species to survive well in deep shade, but lead them to be outcompeted by fast-growing species in high light [17], even irrespective of soil fertility [18]. Thus, the trade-off between plant’s survival in low light and growth in high light occurred. Among tree species with a wide range of morphology and growth potential, slow-growing species having a conservative resource-use strategy are least sensitive to drought [19], although the trade-off between drought tolerance and plant growth is not always significant [20]. Moreover, the C-S-R theory does not similarly employ to all abiotic stresses [21]. A trade-off between plant growth rate and cold hardiness, but not for drought, occurred among plants in 56 families of Douglas-fir [22]. As for salinity, it was hypothesized that the osmotic effect mostly limited the growth of salt-stressed plants, irrespective of the plant’s capacity of excluding salt, resulting in decreased growth rate (biomass) [2]. However, the plant growth potential and salt tolerance trade-off hypothesis was rarely tested.

Among different growth stages (e.g., establishment, flowering and seed filling) in quinoa plants, seedlings are more sensitive to salinity than mature plants [23]. Using five contrasting quinoa cultivars belonging to highland ecotype, we attempted to: (1) ascertain the intraspecific variability (different cultivars) at the establishment stages, based on the morphological (germination, growth) and physio-biochemical responses to salinity, (2) unravel how the underlying morph-physiological determinants vary at the tissue and whole-plant levels in response to salt stress, and, (3) test what the extent of the growth – salt tolerance trade-off existed.

## Results

### Seed germination

**Fig. 1:**
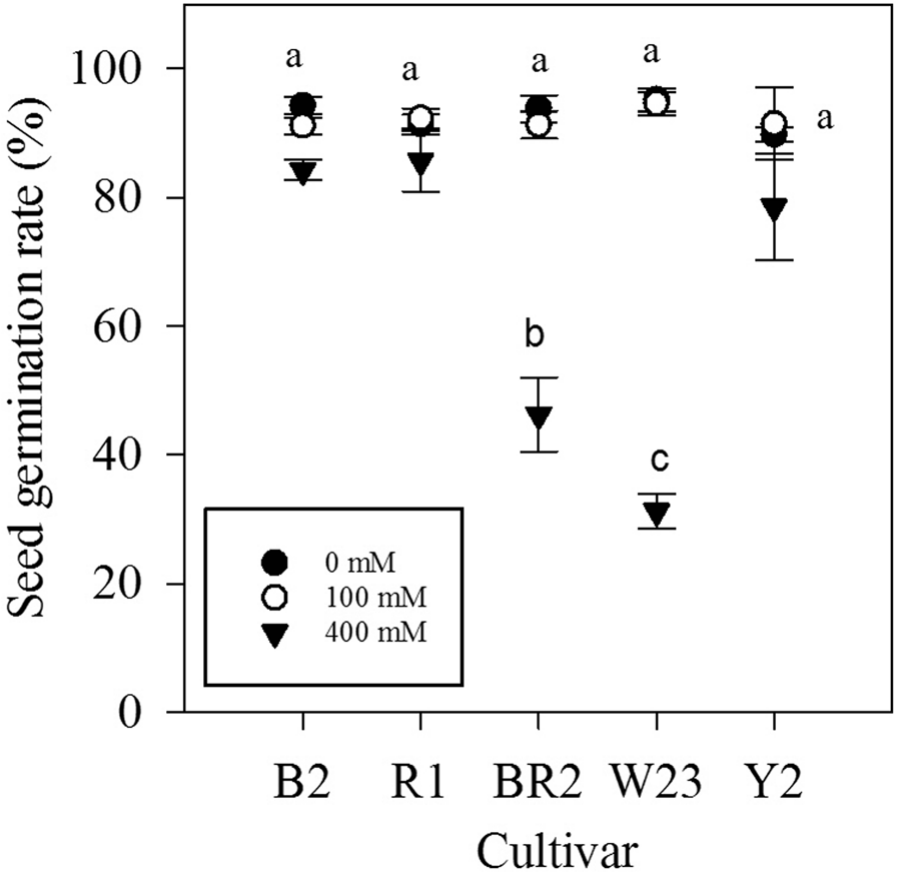
Germination percentage of five quinoa cultivars (five days after sowing) grown on half-strength MS medium supplemented with either 0, 100 or 400 mM NaCl. Each value is the mean ± SD of four replications. Different letters indicate significant differences at *P* < 0.05 level, followed by a Tukey’s post-hoc test

Compared with the control, seed germination did not significantly reduce for each quinoa cultivar in the low salt condition (100 mM NaCl). Whereas, in the high salt condition (400 mM NaCl), germination rates decreased for all cultivars (Fig. 1); sharp inhibition was found in 2 cultivars, i.e., cultivar BR2 and W23.

### Morphological and growth traits

**Fig. 2:**
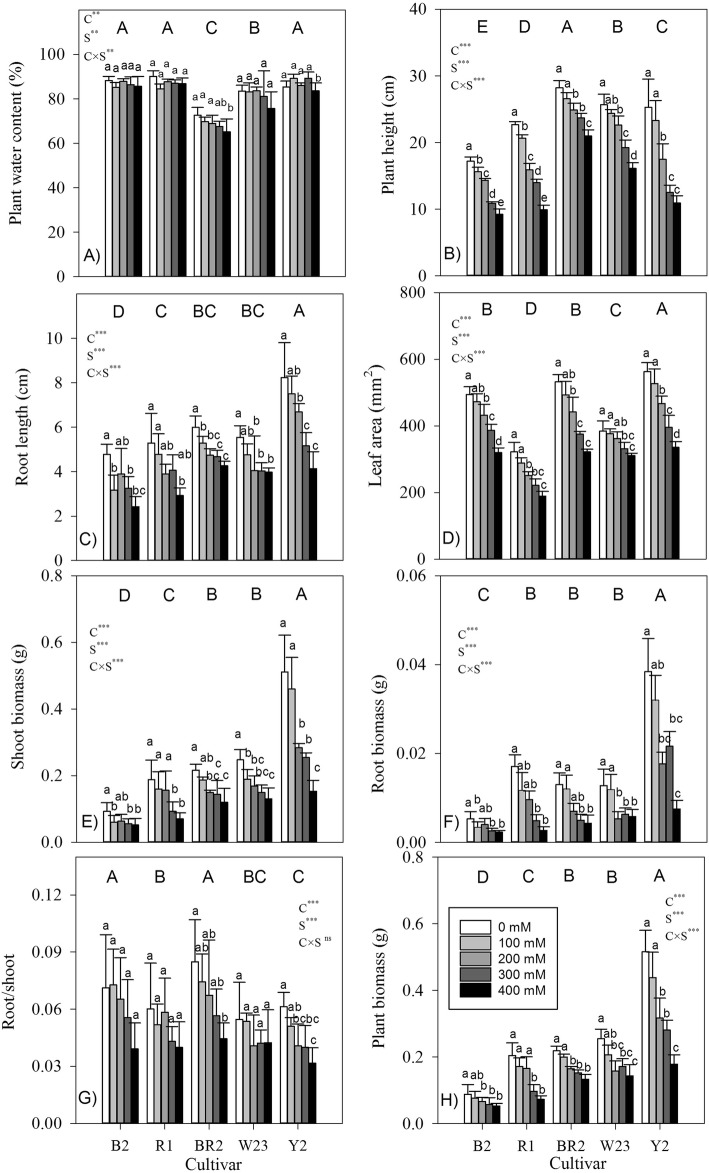
Morphology and growth traits in seedlings of five quinoa cultivars under different salt levels. Different small and capital letters indicate significant differences between the saline levels (S) within each cultivar and between cultivars (C) across different saline levels, respectively, at *P* < 0.05 level. Each value is the mean ± SD of five to six replicate measurements. ns, not significant at *P* > 0.05, * *P* < 0.05, ** *P* < 0.01, *** *P* < 0.001

All plants survived throughout the experimental period (i.e., 100% survival rate), even in the highest salt condition (i.e. 400 mM NaCl). With the lowest value in cultivar BR2 among 5 cultivars, plant water content did not vary greatly in response to salt. Plant height, leaf area, root length, shoot and root biomass, root/shoot ratio and plant biomass generally decreased with increasing salt levels (Fig. 2a-h). Across different salt levels, plant size (biomass) among quinoa cultivars increased in the order of B2 < R1 < BR2 < W23 < Y2; the same pattern occurred even at the control conditions (no salt). Among them, cultivar Y2 had the largest leaf area, root length and biomass (shoot, root and total biomass), but had the medium plant height and the lowest root/shoot ratio; whereas cultivar B2 had the lowest plant height, root length and shoot and root biomass, but had the medium leaf area and the highest root/shoot ratio. Across different salt contents and cultivars, plant biomass was significant (all *P* < 0.05) positively correlated to plant height (r = 0.49), leaf area (r = 0.53), and root length (r = 0.93), but not plant water content (r = 0.098, *P* > 0.05) and root/shoot ratio (r = 0.01, *P* > 0.05).

Except for root/shoot ratio, significant salt × cultivar interactions were found for the measured morphological and growth traits (Fig. 2), indicating that different cultivars varied in the magnitude of their responses to salt. At lower salt levels (< 200 mM NaCl), plant height significantly decreased in cultivars with relatively smaller size (i.e. cultivar B2 and R1), but did not change in cultivars with bigger size (i.e. cultivar BR2, W23 and Y2). Whereas, plant biomass did not change significantly in response to the lowest salt condition (i.e. 100 mM NaCl) for each cultivar, but decreased sharply at higher salt levels (≥200 mM NaCl).

**Fig. 3:**
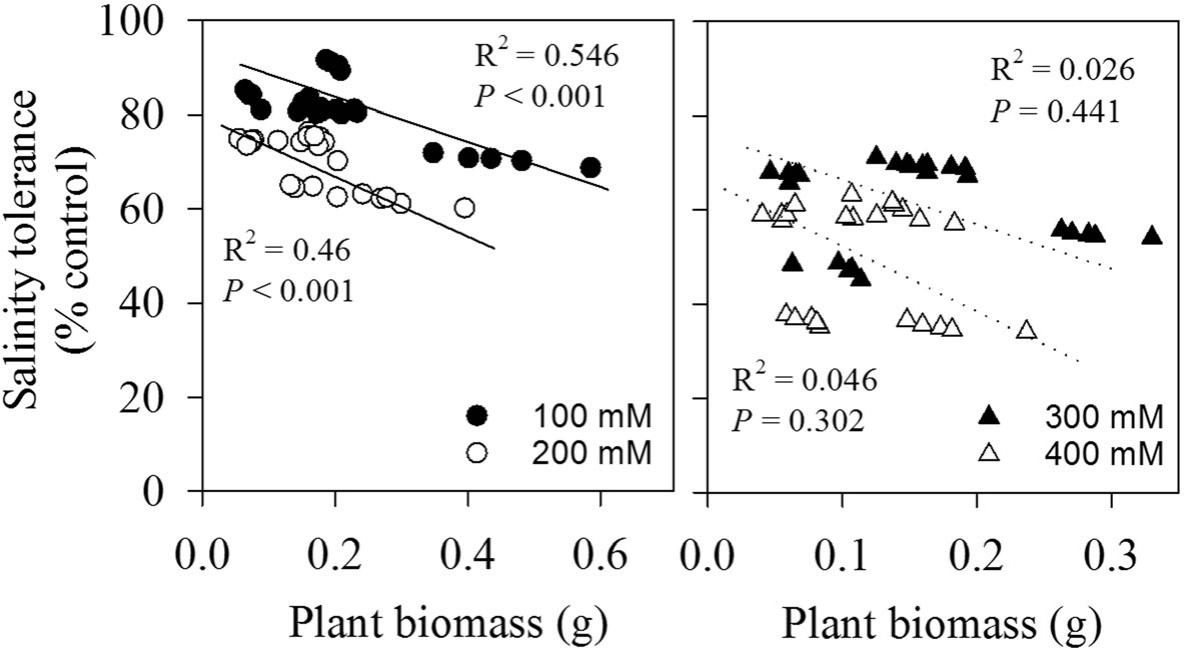
Relationships between plant biomass and salinity tolerance (biomass in salt treatment as % of control) of five quinoa cultivars in each saline level

Relative to the control, ranging from 38.9 to 64.4% among different cultivars, the magnitude of decrease in plant biomass at the highest salt level (400 mM NaCl) generally followed similar pattern as plant size: the decreased percentages in the bigger cultivars (i.e. cultivar W23 and Y2) were more than those in the smaller ones (i.e. cultivar B2 and R1). This was further proved by the negative correlations between plant biomass and overall salinity tolerance (plant biomass in salt treatment as % of control) within each salt level, especially with strong relationship existing at lower salinity levels (< 300 mM NaCl, *P* < 0.05) (Fig. 3).

### Antioxidant enzyme activities and lipid peroxidation in leaves

**Fig. 4:**
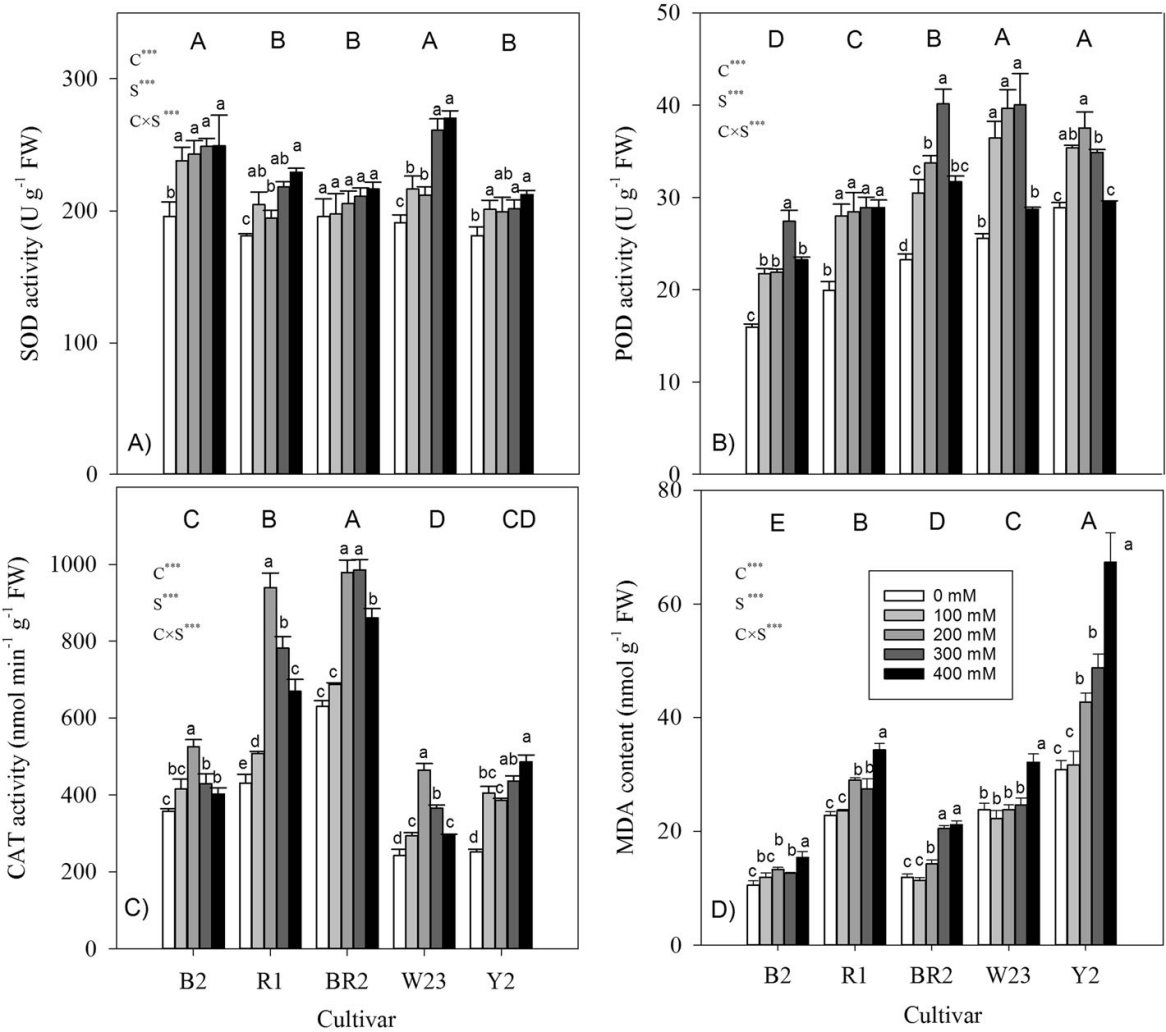
Activities of anti-oxidative enzymes and chlorophyll content in leaves of seedlings of five quinoa cultivars under different saline levels. Different small and capital letters indicate significant differences between the saline levels (S) within each cultivar and between cultivars (C) across different saline levels, respectively, at *P* < 0.05 level. Each value is the mean ± SD of three to four replicate measurements. *** *P* < 0.001

Significant effects of salt, cultivar and salt × cultivar interactions were found for the antioxidant enzyme activities (SOD, POD and CAT) and MDA content in quinoa leaves (Fig. 4). Compared with the control, the activities of each antioxidant enzyme increased, but MDA did not accumulate greatly at the lowest salt level (i.e. 100 mM NaCl). However, with increasing salt levels, SOD activity and MDA content generally increased (Fig. 4a, d), but POD and CAT activities showed non-linear patterns where they first increased but decreased afterwards (Fig. 4b, c). Across different salt levels, POD activities among 5 quinoa cultivars followed similar pattern as plant size, i.e., the bigger cultivars had higher POD activities. But the cultivar with largest size had the highest MDA content, rather than SOD and CAT activities.

### Soluble protein, proline, sugar, and chlorophyll content in leaves

**Fig. 5:**
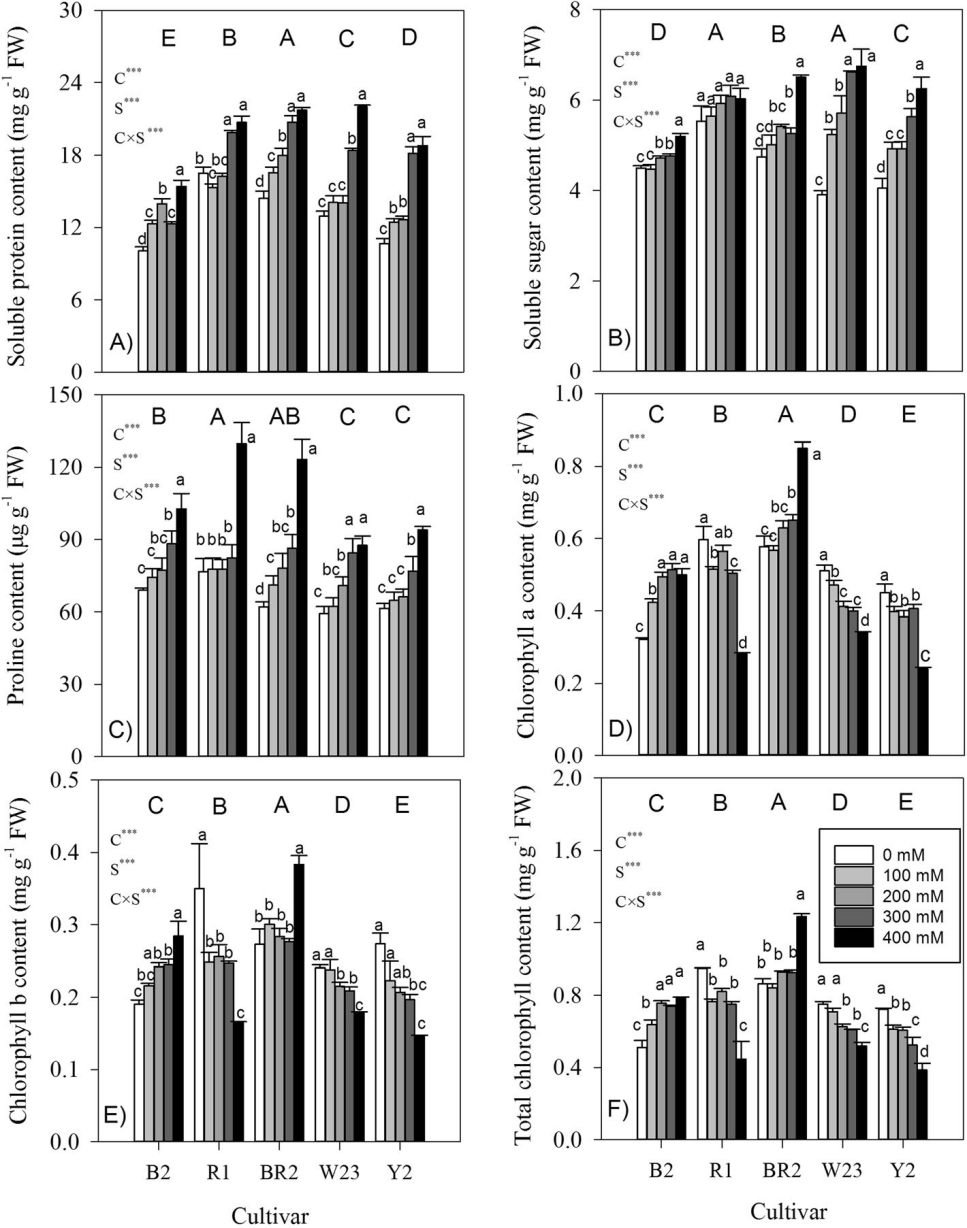
Organic substances and chlorophyll content in leaves of seedlings of five quinoa cultivars under different salt levels. Different small and capital letters indicate significant differences between the saline levels (S) within each cultivar and between cultivars (C) across different saline levels, respectively, at *P* < 0.05 level. Each value is the mean ± SD of three to four replicate measurements. *** *P* < 0.001

Generally, soluble sugar, protein and proline contents increased with increasing salt levels in each cultivar (Fig. 5a-c), but the cultivars differed in the magnitude of responses to salt (significant salt × cultivar interactions, all *P* < 0.001). The significant salt × cultivar interactions for Chl a, Chl b and total Chl contents (Fig. 5d-f) mainly implied that the cultivars differed in their directions of the responses to salt. For example, with increasing salt levels, Chl a, Chl b, and total Chl contents were generally increased in the smallest one (cultivar B2), but decreased in other bigger ones. Across different salt levels, the values of soluble, protein, proline and Chl content did not tightly follow the pattern of plant size. But plant biomass was negatively correlated to each of these organic compounds, although a significant effect was only found in proline (r = *−* 0.529, *P* < 0.05), across different salt contents and cultivars.

### Na^+^ and K^+^ contents and K^+^/Na^+^ ratios in leaves and roots

**Fig. 6:**
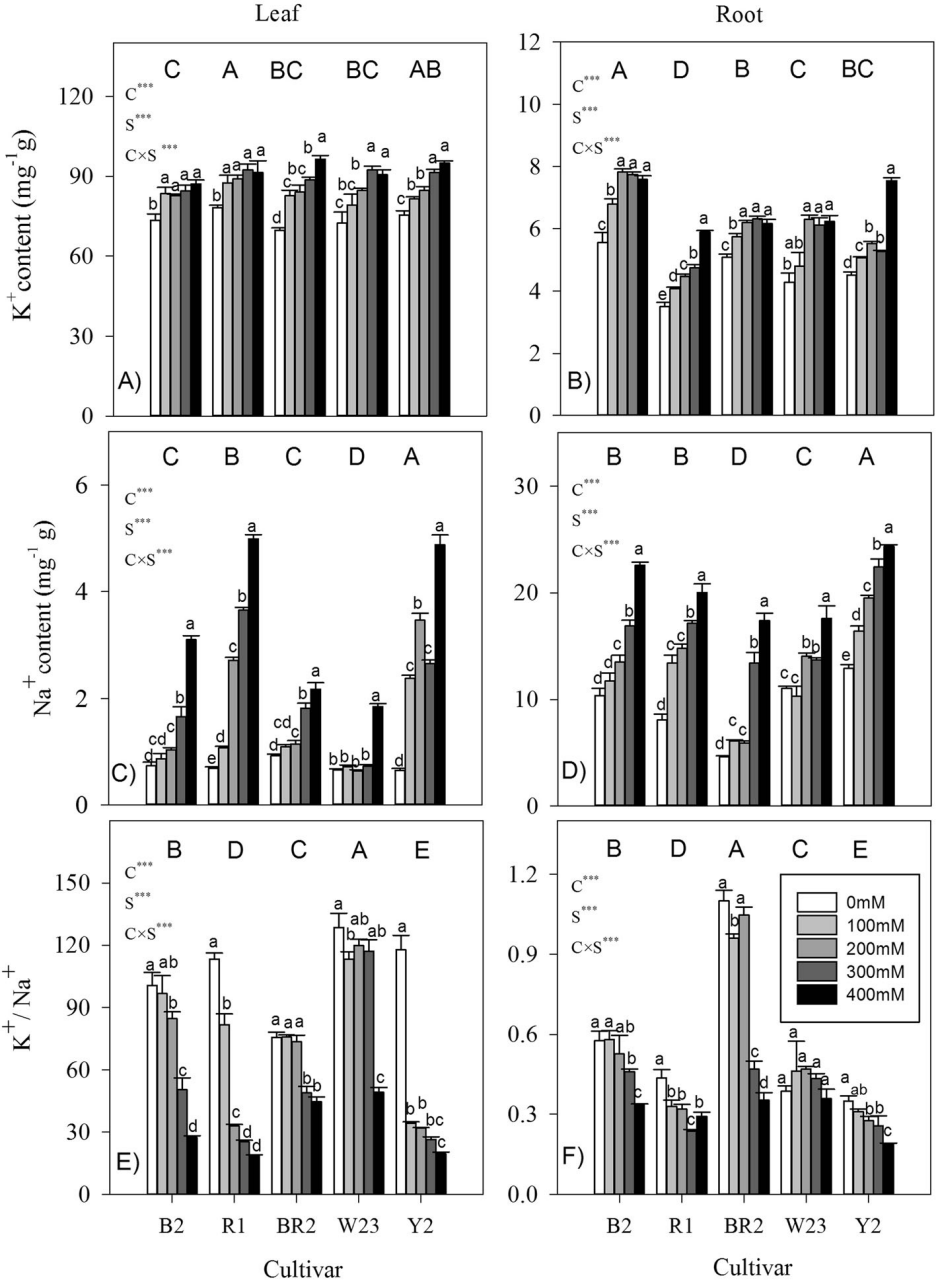
K^+^ and Na^+^ contents and K^+^/Na^+^ ratios in leaves and roots of seedlings of five quinoa cultivars under different saline levels. Different small and capital letters indicate significant differences between the saline levels (S) within each cultivar and between cultivars (C) across different saline levels, respectively, at *P* < 0.05 level. Each value is the mean ± SD of three to four replicate measurements. *** *P* < 0.001

Compared with leaves, roots had much lower values of K^+^ content and K^+^/Na^+^ ratio, but had higher Na^+^ content (Fig. 6, all *P* < 0.001). Thus, regardless of genotype, Na^+^ preferentially accumulated in roots rather than in leaves, and the converse was true for K^+^. Generally, K^+^ and Na^+^ contents in both leaves and roots increased with increasing salt levels in each cultivar (Fig. 6a-d), but K^+^/Na^+^ ratio decreased sharply (Fig 6e, f). Leaf Na^+^ content showed a much greater variation (3.21-fold on average) at the highest salt level relative to the control, compared with that in root (1.65-fold on average). The reverse occurred for K^+^ contents (on average 1.22 vs. 1.73-fold for leaf and root, respectively). Among cultivars across different salt levels, cultivar Y2 had the highest Na^+^ contents and lowest K^+^/Na^+^ ratio in both leaves and roots. But the values of K^+^ and Na^+^ contents and K^+^/Na^+^ ratio in leaves or roots did not tightly follow the pattern of plant size. There were negative trends between plant biomass, and, K^+^ and Na^+^ contents in both leaves and roots across different salt contents and cultivars (r = − 0.074--0.0421, all *P* > 0.05), although not significantly.

### Relationships between salinity tolerance with variables

**Fig. 7:**
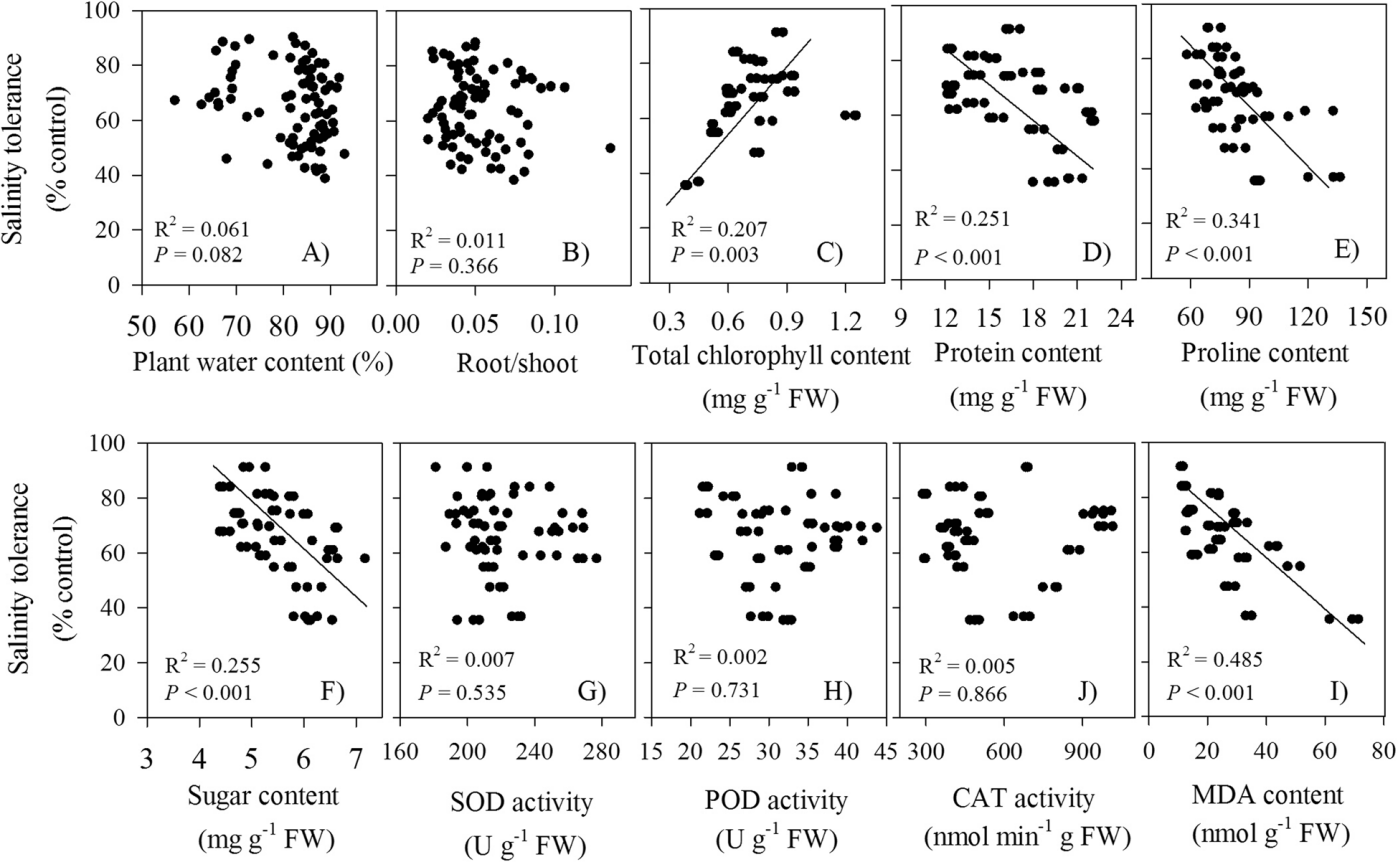
Relationships between salinity tolerance (biomass in salt treatment as % of control) and the morph-physiological traits across different cultivars and saline levels

**Fig. 8:**
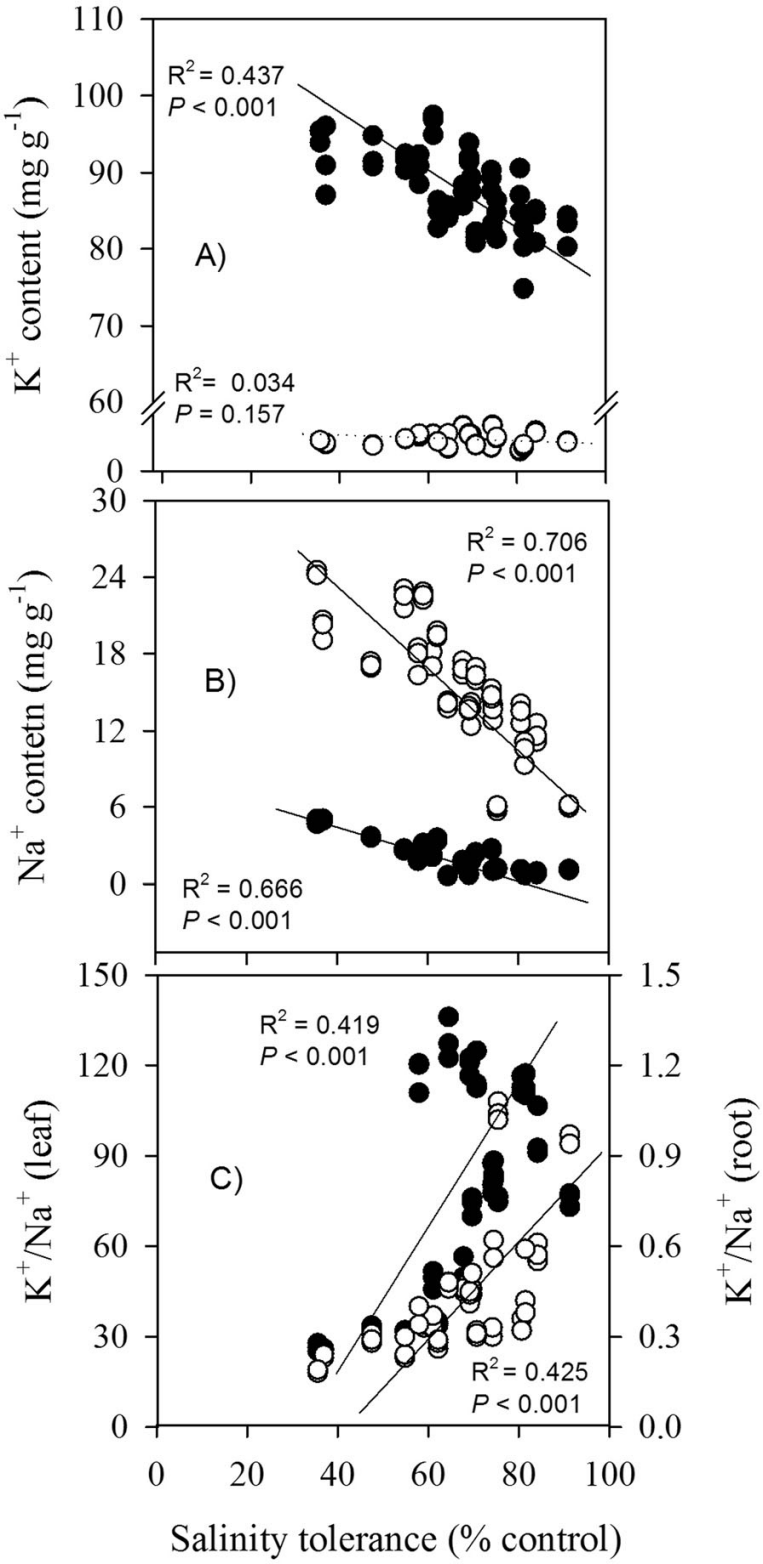
Relationships between salinity tolerance (biomass in salt treatment as % of control) and ion contents and K^+^/Na^+^ ratio in leaves (●) or roots (○) across different cultivars and saline levels

Across different cultivars and salt levels, without close relationships with plant water content, root/shoot ratio and antioxidant enzyme activities (SOD, POD, or CAT), salinity tolerance was significant negatively correlated with leaf proline, protein, sugar and MDA contents, respectively, but was positively correlated with total Chl contents (Fig. 7). For inorganic ions, except for root K^+^, salinity tolerance was significant negatively correlated with K^+^ and Na^+^ contents but positively correlated with K^+^/Na^+^ ratio in leaves or roots (Fig. 8). Among the traits assessed, Na^+^ contents in leaves and roots had the strongest determinant of salinity tolerance (R^2^ = 0.666–0.706). Relatively, the correlations with leaf organic solutes (proline, protein, or sugar) and salinity tolerance were much lower (R^2^ = 0.251–0.341).

## Discussion

### Salt tolerance at seed germination is not consistent with that in the seedling stage

Seed germination often occurs on soil surface in saline environment, exposing the seeds and seedlings to higher salt levels than older plants. Some halophytes are even relatively more sensitive to salinity during the establishment stage [24, 25]. In our study, seed germination of five highland quinoa cultivars did not reduce at low salinity level (100 mM NaCl), but two of them showed significant decrease at high salinity level (400 mM NaCl) (Fig. 1). In a previous study with four lowland quinoa genotypes in Chile, only one (BO78) revealed significant reduction in seed germination rate at high salinity level (300 mM NaCl) [13]; while for a less salt-tolerant cultivar selected in Denmark (cv. 5206), inhibitory effect was obvious only at high salinity levels (> 400 mM NaCl) [12]. Across our studied highland cultivars, tolerance at high salinity level during germination was not consistent with relative plant growth (biomass) reduction. Taken together, it can be said that, at germination, salt tolerance of quinoa largely depends on its genotype and/or ecotype. Since these two processes are governed by different mechanisms [26, 27]; salt tolerance between seed germination and seedling is not necessarily correlated.

### Small plants are more tolerant to salt

Plant water content or leaf water content, though widely employed, is not a strong indicator of turgor in salt-stressed plants undergoing osmotic adjustment. This is due to the fact that, greater solute content of cells at higher salinity largely results from ion (e.g. Na^+^ and Cl^−^) and organic solutes accumulation but not from water loss, especially in halophytes [1, 2]. In response to the increased salinity levels, plant water content did not vary greatly, but plant height, leaf area, and root length decreased sharply (Fig. 2a-f) (c.f [26].). Compared with the control (i.e. no salinity), plant height of some Peruvian quinoa accessions reduced under salinity, whereas some of them even indicated taller growth [25]. Rather than developing deep and dense root system to ameliorate the negative effects of drought to “find” more water [26], highland quinoa decreased root growth and elongation (Fig. 2c,f), thus, avoiding excessive uptake of Na^+^ and Cl^−^ and also preventing an escalation in salt concentration in soils [2]. Increase, decrease and maintenance of root/shoot ratio were previously found to be a genotype-dependent response in quinoa [13]. Decreased root/shoot ratio with increasing salinity levels (Fig. 2e) indicated stronger influence of salt on root biomass than shoot biomass [24, 26]. But the plant’s early morphological response via adjustment of root and shoot biomass did not play an important role in salt stress because of absence of any close relationship between root/shoot ratio and salt tolerance (Fig. 7b). Reduction in leaf area (Fig. 2d), the most obvious avoidance mechanism to cope up with salt stress for many crops including halophytes [2, 26], resulted in a consequent functional reduction in assimilatory unit of plants and decreased water use by the plant, thus conserving soil moisture.

We did not find significant change in plant biomass between the control and the lowest salinity level (100 mM NaCl) in each studied cultivar, although salinity stress progressively reduced shoot, root, and total biomass (Fig. 2e, f, h). This contrasted with previous results where, in some quinoa genotypes, optimal growth was achieved at intermediate salinity levels (i.e. 100–200 mM) [10, 12, 27]. Slower growth at initial stage under stress conditions might be an adaptive response of plants to survive that allows them to store resources, repair damaged structures, and restart physiological functions [15, 16]. A negative trend between plant biomass and salt tolerance within each salt level (Fig. 3) indicated that quinoa cultivars with smaller size (biomass) are more salt tolerant, especially with strong relationship existing at lower salinity levels (< 300 mM NaCl). Compared with the high salt levels, genetic variation in Na^+^ exclusion may contribute to greater tolerance at moderate salinity conditions where leaf Na^+^ content is below toxic level. Thus, our results provided some support for the presumed tradeoff between seedling’s growth potential and salt tolerance at the intraspecific level of highland quinoa, as stress adaptation is costly. This “trade-off” was previously observed in 2 quinoa cultivars having highly contrasting origin with respect to salinity: the salt-tolerant cultivar Utusaya belonging to Salares ecotype, and, the less salt-tolerant Danish-bred cultivar Titicaca [25]. Even in adult plants, a trade-off between salt-stress adaptation and plant growth was found among two coast-lowland quinoa landraces (VI-1, Villarrica) and a salt-tolerant Salares cultivar (R49) [3]. However, in response to drought, the relationship between plant’s growth potential and drought tolerance across eight desert grasses may be somehow explained by differential response of plants on soil water content, rather than the inter-specific differences in drought tolerance [20].

Chlorophyll content generally increased in the most salt-tolerant quinoa cultivar (i.e. B2), but decreased in others (Fig. 5f). It was also reported that chlorophyll content significantly decreased in a less salt-tolerant cultivar (Titicaca) but increased in a salt-tolerant cultivar (Utusaya) [26]. Positive relationship between chlorophyll content and salt tolerance (Fig. 7c) may be considered as a compensatory mechanism aimed to protect quinoa for their inability to efficiently exclude Na^+^ from uptake into leaves and enhance CO_2_ fixation, with less growth penalty.

### The measured antioxidant enzymes may not be a key element for salt tolerance

Increased activities of antioxidant enzymes (like SOD, CAT, POD) might mitigate effects of oxidative damage that often characterize plant responses to stress [28]. SOD dismutates O_2_^·–^ into H_2_O_2_; whereas, presenting in the peroxisomes, CAT mainly catalyzes decomposition of H_2_O_2_ into water and oxygen, and POD mainly scavenge H_2_O_2_ in chloroplasts. As one of the first line of defense against oxidative stress, SOD activity had no positive or negative correlation with salinity tolerance in glycophytes [29, 30]. But halophytes may show an exceptional ability to utilize the immediate stress-induced SOD production, thus sending stress signals through H_2_O_2_ formation to protect themselves from adverse conditions [31, 32]. It was found that, the enhanced antioxidant enzyme activities in quinoa cultivar Titicaca could be one of the factors responsible for salt tolerance, although having lower activity than its counterpart in cultivar Utusaya [24, 33]. Previously, a controversial increase and decrease in CAT and POD activity under salt and drought stress in Pichaman genotype of quinoa was found; that might be attributed to both genetic variation and variances in kinetics of stress development [25]. At the lowest salt level, antioxidant enzymes counteracted the adverse effects of O_2_^−^ as well as H_2_O_2_, since the activities of antioxidant enzymes (SOD, POD and CAT) increased but MDA did not accumulate greatly in leaves (Fig. 4). Consequently, a degree of oxidative damage at cellular level was mitigated. Antioxidant system works in a coordinated manner, SOD activity producing cytotoxic H_2_O_2_, while itself being neutralized by superoxide [28]. Whereas at higher salt levels (> 100 mM NaCl), SOD activities continued to increase; POD and CAT activities first increased and then decreased, and, MDA also accumulated significantly (Fig. 4). Significant increase in leaf MDA content at higher salt levels in all cultivars can be evidence of increased damage to membranes due to generation of more reactive oxygen species (ROS). The plausible answer in our study might be the important role played by H_2_O_2_ as a second messenger, triggering cascades of adaptive responses at both physiological and genetic levels, where rapid conversion of O_2_^−^ to H_2_O_2_ by SOD is essential for generating early defense signal in halophytes [2, 31]. Having enough SOD ‘in stock’, quinoa plants rapidly induce H_2_O_2_ levels, rather than detoxifying by SOD, which give them a certain adaptive advantage. Compared with *Arabidopsis thaliana* (a glycophyte), it was clear that *Cakile maritime* (a halophyte) could quickly send stress signals through H_2_O_2_ and had an efficient antioxidant mechanism to scavenge it upon completion of signaling [34]. It was also reported that high salt-induced reduction of CAT activity might be explained by the requirement to increase H_2_O_2_ levels to be used in stress signal transduction [28]. In addition, H_2_O_2_ played an important role in the regulation of K^+^/Na^+^ homeostasis and increased resistance to salt stress in callus tissue of salt-tolerant *Populus euphratica* [35]. Thus, it will be interesting to evaluate the actual role (signaling or scavenging) of CAT and POD in halophytes like quinoa.

Salinity tolerance was not closely related to antioxidant enzyme activities (SOD, POD, or CAT) across different salt levels and quinoa cultivars (Fig. 7g-j), similar to the genotypes of barley [29] and some halophytes (e.g. *Atriplex lentiformis*) [32]. The antioxidant enzymes, therefore, may not be a key element for salt tolerance in highland quinoa. As a halophyte, quinoa possesses efficient means to handle salt load (e.g. Na^+^ exclusion from the cytosol) without the requirement of a high level of antioxidant activity, as they simply prevent excessive ROS formation in salt stress conditions [10, 32]. Genetic differences in salinity tolerance are not necessarily owe to differences in the ability to detoxify ROS. Additionally, although halophytes may use the antioxidant machinery more efficiently than glycophytes, non-enzymatic antioxidants, e.g., glutathione reductase and glutathione, and compatible solutes (mannitol, myo-inositol, proline and glycine-betaine, etc.) present in quinoa can play a crucial role [3, 27].

### Accumulation of organic osmolytes may be adaptive

The contents of organic solutes in leaves (i.e. soluble sugar, protein and proline) generally increased with increasing salt levels for each studied cultivar (Fig. 5a-c), which was also widely reported in some quinoa genotypes in response to drought and salt stress [23, 36]. Accumulation of soluble sugars and other compatible solutes (e.g. proline) not only allow plants to decrease osmotic potential and maintain the cellular turgidity necessary for cell expansion under salinity stress conditions (osmotic adjustment) but also act as osmoprotectants, helping the cells to protect and maintain membrane integrity [4, 30]. The enhanced production of total soluble sugars in quinoa seedlings was presumed to adjust osmotically to saline environment [27]. As an osmolyte that is frequently found in plants subjected to drought and salinity conditions [32, 37], the increased sugar content in quinoa might be due to salinity stress, which was further supported by high activities of soluble acid invertase and sucrose–phosphate synthase in salt-stressed quinoa seedlings [13]. Excessive high content of sugar may, however, inhibit photosynthesis by a feedback mechanism, causing a reduction of leaf development and hence plant growth; similar to the negative correlation observed here between sugar contents and salt tolerance (Fig. 7f). Carbohydrates act as an active carbon sink prior to plant growth; an increase in carbon storage and decrease in plant growth could be a plastic or evolutionary response to carbon-limiting conditions [16, 37].

Apart from osmotic adjustment, proline is considered to perform multiple antioxidant functions, thereby ensuring membrane stabilization, and protection of photosynthetic machinery against oxidative stress in developing leaves [1]. However, there were inconsistent results about whether proline accumulation serves as a mechanism to tolerate salinity or it was a negative consequence of salinity [38, 39]. For instance, in rice, some authors suggested that salt-stress induced proline accumulation was related to the degree of salt tolerance [40]. Whereas others argued that proline accumulation in salt-sensitive rice was a symptom of salt stress injury reflecting poor performance and greater damage, resulting from the increased ornithine δ-aminotransferase activity and the endogenous pool of its precursor glutamate [39]. Proline enhancement occurred at the onset of the lower salinity level in quinoa (Fig. 5c), supporting the hypothesis that this accumulation is initially a reaction to salt stress damage [4, 38]. However, the relationship between the ability of proline accumulation on stress imposition and specie’s stress tolerance is not very clear, although plant species differed greatly in the amount of proline responded to stress. For instance, cell elongation in roots in drought-stressed maize was maintained in cells that accumulate proline; hence, proline accumulation was not associated with reduced growth [41]. The inverse relationship between proline content and salt tolerance in highland quinoas (Fig. 7e) may be related to the fact that the synthesis of proline consumed high nitrogen source and energy, at the cost of plant growth. This result in our studied highland quinoas contrasted partly with that in four lowland quinoa genotypes, where the most salt tolerant genotype showed the highest increment in proline content upon salt conditions [13], but consistent with salt-sensitive barley [4] and sorghum [38]. Even at the cellular and, ultimately, organismal level, it is clear that the level of proline accumulation and the amount of growth are inversely correlated under salt conditions; being a part of adaptation process, proline may act as a signaling molecule capable of activating multiple responses [42]. Being demonstrated in a range of halophytic species, the beneficial effect of adaptive proline accumulation is that it served as an osmolyte and protection for quinoa plants under salt-stress conditions, rather than enhancing plant growth (salt tolerance) [4]. Whereas, more work are needed to understand the relationship between proline accumulation, stress adaptation, and control of plant growth and development in quinoa, especially in the field.

### K^+^/Na^+^ ratio is positively correlated to salt tolerance

Leaves had much lower Na^+^ content, but higher K^+^ content than roots (Fig. 6a-d). As a whole-body response in adaptation to salt, plants preserve Na^+^ in the roots, due to their relatively higher tolerance to ion toxicity than leaves, and restrict Na^+^ flux to the shoot and leaves. Compared with roots, much lower leaf Na^+^ content suggested that these quinoa cultivars are “Na^+^ excluders” [14]. Na^+^ exclusion from cells and compartmentalization and safe lock of excessive Na^+^ in the vacuole of leaves in quinoa are important protective ways in response to salt-induced ion toxicity at the cellular level [43]. Moreover, quinoa plants tolerate saline conditions by dumping excess salt into specialised epidermal bladder cells on the leaves, which constitutively sequester and excrete them actively from metabolically active cells [10, 43]. It is now becoming clear that the inward-rectifier high-affinity K^+^ transporters (HKT1.2) is the underlying one-way accumulation system playing a key role for Na^+^ load into bladder cells in quinoa [11]. This surplus salt, mainly Na^+^, compartmentalize from the leaf blades into the bladder hairs located on the leaf surfaces, from where it can be washed off by rain.

Salt increased K^+^ and Na^+^ contents in both leaves and root (Fig. 6a-d); the accumulated inorganic ions in tissues thereby enable quinoa plants to maintain cell turgor and to reduce transpiration under salt stressed conditions, via adjustment of water potential [2, 12]. Potassium is released from roots to xylem for transport to the leaves; increased leaf K^+^ content could be attributed to an exchange between Na^+^ and K^+^ in the proximal part of roots [44]. Our results are consistent with the previous works that accumulation of inorganic ion and organic solutes occurred in salt-stressed quinoa plants [5, 12, 25], although inorganic osmoregulation had the strongest contribution to osmotic adjustment (ca. 90%) [12]. Higher K^+^ content with increasing salinity levels indicated that K^+^ uptake was enhanced by Na^+^ supply. This seems to be counterintuitive, as the two ions competing for major binding sites in the important metabolic processes in cytoplasm and K deficiency always occurred when imposed by salinity [1]. In other work on barley [45] and some halophytes, e.g. quinoa [5, 10, 12], K^+^ accumulated in some tissues and contributed more efficiently in osmotic adjustment in cells of leaves under high salinity conditions. In the former work, it was interpreted that a higher demand is for “free” K^+^, not “structural” K^+^, in order to osmotically adjust and support leaf expansion. We only measured total ion content but not fluxes themselves, therefore, it is not possible to confirm such possibilities from our study. Essential for a range of physiological processes in response to salt stress, leaf K^+^ loss may activate an amount of caspase-like proteases triggering programmed cell death, and thus enhance leaf senescence [46]. In this context, the quinoa plants’ ability to increase K^+^ uptake and retention in plant tissues (especially in leaf) is a part of its extraordinary salinity tolerance (c.f [10].).

Salinity tolerance was significantly negatively correlated with K^+^ and Na^+^ contents in leaves or roots, except for root K^+^ (Fig. 8a,b). Compared with the organic osmolytes (i.e. protein, sugars, and proline) in leaves, inorganic ions showed higher correlations with salinity tolerance (Figs. 7, 8), indicating that they probably made larger contribution in osmoregulation. Relative to organic osmolytes with high energy cost of de novo synthesis [47], it is much more advantageous and metabolically cheaper for plants to use inorganic solutes for osmotic adjustment, assuming it will not interfere with cell metabolism. But strikingly, in 11 genotypes of “Na^+^ includer”, a positive correlation was observed between the accumulated Na^+^ amount and plant’s salinity tolerance [14]. An inverse relationship between leaf Na^+^ accumulation and salinity tolerance often occurred when different genotypes within a species are compared, but this is not the case in inter-specific comparison, such as in wheat and barley [1]. On the other hand, the general assumption of increased levels of K^+^ to mitigate salt stress is probably oversimplified. In NaCl-treated *Arabidopsis* plants, over-accumulation of Na^+^ and K^+^ triggered growth reduction, through stomatal regulation or systemic stress responses, rather than Na^+^ toxicity and water deficit [2]. The negative correlation between leaf Na^+^ content and plant salinity tolerance suggested that the major mechanism contributing to salinity tolerance was to exclude salt from their leaves, rather than vacuolar Na^+^ sequestration. At the whole-plant level, protecting young leaves from excessive Na^+^ amounts has long been considered as a key attribute of Na^+^ compartmentalization in many species [1, 43]. Quinoa is no exception.

In addition, maintenance of ion (especially K^+^) homeostasis is essential for ionic and pH homeostasis, enzyme activities, and cytosolic K^+^ attributed to the plant adaptive responses to a broad range of abiotic stresses [1, 44]. Quinoa plants accumulated more Na^+^ than K^+^ under salinity stress, as the K^+^/Na^+^ ratios in both leaves and roots decreased with increasing salinity levels (Fig. 6e,f). Being one of the most important cations for plant growth, K^+^ is required as an enzyme cofactor and as a vacuolar osmoticum. The catalytic sites normally bind the essential K^+^ and maintain a high cytosolic K^+^/Na^+^ ratio to enhance salt tolerance [44]. Similarities between Na^+^ and K^+^ lead to competition during transport in these sites. The K^+^/Na^+^ ratio in leaves or roots was positively correlated with salinity tolerance (Fig. 8c), which was also found in drought resistant quinoa [36] and, commonly, in glycophytes [1]. Thus, maintenance of ion homeostasis is critical for salt tolerance of our studied highland quinoa plants. As an index of salinity tolerance, K^+^/Na^+^ ratio in the vegetative tissues (i.e. leaf or root), therefore, can be used as a convenient selection criterion in the breeding of highland quinoa cultivars.

## Conclusions

Quinoa cultivars belonging to highland ecotype revealed substantial variations in plant size (biomass) and salinity tolerance, where salinity tolerance of quinoa was negatively correlated with plant size. The interactions of cultivar and salt were found for all measured plant traits, except for shoot/root ratio. With increasing salt levels, accumulation of organic (protein, sugars, and proline) and inorganic (K^+^, Na^+^) substances in quinoa plants might be a reflection of the energetic cost associated with osmotic adjustment. During resource limitation under salt stress conditions, active synthesis of these compounds may enable plants to survive and recover from stress, at the expense of plant growth as those solutes are no longer available for cell wall and protein synthesis [1, 3, 37]. Leaf osmoregulation, K^+^ retention, Na^+^ exclusion, and ion homeostasis are the main physiological mechanisms, rather than leaf antioxidant regulations, conferring salinity tolerance to these cultivars. The C-S-R theory is applicable to highland quinoa cultivars, since an apparent trade-off between growth and salt tolerance existed. This trade-off creates a practical challenge to instill resilience into domesticated populations without compromising yields, although plant breeders generally wish to identify the fast-growing and stress-tolerant genotypes.

## Methods

### Plant materials and experimental design

Seeds of 5 cultivars of *C. quinoa*, all belonging to the highlands ecotype, were obtained from the quinoa gene bank of Universidad Nacional del Altiplano, Peru. Brief information of the studied cultivars has been described in Table 1. Mature seeds were sown on dishes (15 cm) containing autoclaved half-strength MS medium and 0.6% (w/v) Phytagel (Sigma-Aldrich). Plates (5 per treatment) contain 40 seeds each in growth chambers at 25 ± 0.5 °C under a 14/10 h light/dark photoperiod (150 μmol m^2^s^− 1^). To determine seed germination in response to salt, seeds were sown on media containing 0 (control), 100 and 400 mM NaCl. As the fact that quinoa’s seeds germinated unusually fast [23], percentage germination was measured on the 5th day after sowing.

**Table 1:** Origin and characters of the quinoa cultivars used in this experiment

Genotype	Local code	Origin	Photoperiod sensitivity	Other characters
# 23	B2	Peru (Puno)	Neutral day	White stem and inflorescence, green leaves, less tolerant to frost. Late maturing. Black seeds; seed weight: 0.00297 g per seed.
# 4	R1	Peru (Puno)	Short-neutral day	Red stem and inflorescence, red young leaves, tolerant to frost and downy mildew. Early maturing. Red seeds; seed weight: 0.00272 g per seed.
# 24	BR2	Peru (Puno)	Short day	White stem. Maturing medium-early to late. Brown seeds; seed weight: 0.00349 g per seed.
# 32	W23	Peru (Casco)	Short day	White stem and inflorescence; tolerant to frost and drought. Early maturing. White seeds; seed weight: 0.00292 g per seed.
# 14	Y2	Bolivia (southern altiplano)	Short day	Yellow stem. Panicle colored from white to yellow. Late maturing. Yellow seeds; seed weight: 0.00486 g per seed.

For seedling growth, the experiment was carried out in a glass greenhouse at a temperature between 19 °C and 25 °C and an average humidity of ca. 75% in Xishuangbanna Tropical Botanical Garden (21°56′N, 101°15′E), Chinese Academy of Sciences. The quinoa plants were grown in 5-L plastic pots containing 2.0 kg of a mixture (v/v) of 80% Novarbo Substrates (Novarbo Oy, Finland) and 20% sandy soil. Five to six surface sterilised seeds per pot were sown. When seedlings reached the two-leaf stage, plants were thinned to two with uniform growth per pot. Plants were randomly rearranged twice a week throughout the experimental period, to ensure uniform growth conditions. When plants had four to five well-expanded leaves, they were watered with Hoagland’s solution 2 times per week, maintaining at field capacity until the beginning of the stress treatment. Afterwards, plants of each cultivar were divided into 5 groups: plants were watered every 2 days with 200 mL Hoagland’s solution supplemented with 5 different NaCl contents [i.e., 0 (control), 100, 200, 300, and 400 mM]. Salt stress treatment was imposed for 21 days; 8 replicates (pots) were used for each treatment within each cultivar.

### Morphology, growth and chlorophyll parameters

At the end of the experiment, seedlings were harvested and the whole-plant fresh weight (FW) was immediately assessed. Subsequently, plant height and root length were measured. All leaf samples per individual plant were measured with a LI-3000C leaf area meter (LI-Cor Inc., Lincoln, NE, USA). After that, plant samples were dried at 70 °C for 48 h to constant weight to obtain plant biomass (dry weight, DW) per individual plant; and dry weights of leaves, stems, and roots were separately determined. Plant water content (%) was calculated as: (FW-DW)/FW × 100 at individual plant level. For leaf physiological and biochemical measurements, the fully-expanded, young leaves were used. Leaf chlorophylls (Chl) were extracted in 80% acetone and absorbance at 663 and 645 nm were measured. Chl a, Chl b and total Chl contents were then calculated. Morphological and growth measurements were made on 5–7 plants, while physiological and biochemical measurements were made on one leaf per plant from 3 to 4 plants per cultivar per treatment.

### Measurements of antioxidant enzyme activities and lipid peroxidation

For antioxidant enzyme extractions, 0.5 g of fresh leaves was homogenized with 50-mM potassium phosphate buffer (pH 7.8), containing 1 mM EDTA, 3 mM 2-mercaptoethanol, and 2% (w/v) polyvinyl-poly-pyrrolidone. The filtered homogenate was then centrifuged at 15,000 *g* for 30 min at 4 °C, and the resulting supernatant was used to evaluate the activity of superoxide dismutase (SOD, EC 1.15.1.1), catalase (CAT, EC 1.11.1.6), and peroxidase (POD, EC 1.11.1.7). All enzyme activities were measured at 25 °C by an UV-B spectrophotometer (UV-B 2501, Shimadzu, Japan). SOD activity was assayed by monitoring the inhibition of photochemical reduction of nitro blue tetrazolium (NBT) using the method of Beauchamp and Fridovich (1971) [48]. One unit of SOD activity was defined as the amount of enzyme required to cause 50% inhibition of NBT reduction. The POD activity was determined as described by Hemeda and Kelin (1990) [49] using guaiacol as a substrate. One unit of POD activity was defined as the amount of enzyme that increased the absorbance at 470 nm by 0.001 absorbance unit per min. Catalase activity was estimated by monitoring the disappearance of H_2_O_2_ at 240 nm [50]. Membrane lipid peroxidation was recorded by the spectrophotometric determination of malondialdehyde using thiobarbituric acid.

### Determinations of organic and inorganic solutes

The soluble protein content was measured as described by Bradford (1976) [51]; bovine serum albumin was used as a standard. Total soluble sugar was estimated from the glucose standard curve according to Dubois et al. (1956) [52]. Free proline content was determined according to Bates et al. (1973) [53] with minor modifications. The proline content was determined from a standard curve of L-proline.

For the measurements of Na^+^ and K^+^ contents, dry-ashed tissues (leaf and root) were wet digested using HNO_3_:HClO_4_ (7:3 v/v). The contents of Na^+^ and K^+^ were determined using an inductively coupled plasma atomic emission spectrometry (iCAP6300, Thermo Fisher Scientific, USA).

### Statistical analyses

Data was analyzed with a two-way ANOVA for each variable, with cultivar (C) and salinity(S) as main fixed factors plus an C × S interaction term, followed by a Tukey HSD post hoc test within cultivars or salinity levels. We tested the assumptions of an ANOVA prior to analyses. Transformation was applied before statistical analysis being performed, when necessary. Pearson’s correlations were used to analyzed the correlations amongst traits. All statistical analyses were conducted using SPSS version 21.0 (SPSS, Chicago, IL, USA).

## Data Availability

The datasets generated during the current study are available from the first author on reasonable request.

## Abbreviations

CAT: Catalase
Chl: Chlorophylls
MDA: Malondialdehyde
POD: Peroxidase
SOD: Superoxide dismutase
